# Changes in Gut Microorganism in Patients with Positive Immune Antibody-Associated Recurrent Abortion

**DOI:** 10.1155/2020/4673250

**Published:** 2020-09-18

**Authors:** Min Jin, Dong Li, Rui Ji, Wen Liu, XiaoFei Xu, Xin Feng

**Affiliations:** ^1^Department of Anesthesiology, Qilu Hospital, Shandong University, Jinan, Shandong 250012, China; ^2^Cryomedicine Laboratory, Qilu Hospital, Shandong University, Jinan, Shandong 250012, China; ^3^Department of Gastroenterology, Qilu Hospital, Shandong University, Jinan, Shandong 250012, China; ^4^Department of Obstetrics and Gynecology, Qilu Hospital, Shandong University, Jinan, Shandong 250012, China; ^5^Qilu Hospital, Shandong University, Jinan, Shandong 250012, China

## Abstract

**Background:**

This study is aimed at analyzing the changes in gut microorganism of patients with positive immune antibody-associated recurrent abortion using the 16s rRNA gene sequencing microbiome assay.

**Methods:**

The fecal samples from 20 recurrent abortion women with positive immune antibody (positive group) and 20 with negative immune antibody (negative group) were collected. After 16s rRNA gene sequencing, the obtained raw reads underwent quality filtering to obtain the clean tags and then classified into microbial genomes. All effective tags were clustered into operational taxonomic units (OTUs), and the representative sequence was selected for the annotation of taxonomic information, followed by alpha and beta diversity analyses.

**Results:**

A total of 43,116 OTUs were obtained in all 40 samples. *Bacteroides* had the highest relative abundance in the positive group. In the negative group, *Bacteroides*, *Erysipelotrichaceae*_UCG-003, *Faecalibacterium*, and *Prevotella_9* had high relative abundance. Alpha diversity analysis results showed that the community richness, community diversity, and phylogenetic diversity in the positive group were higher than that in the negative group. *Prevotella*_9, *Enterococcus*, *Megasphaera*, and *Anaerostipes* presented significant differences between negative and positive groups.

**Conclusion:**

The present study for the first time investigated the gut microbiome involved in positive immune antibody-associated recurrent abortion via the 16s rRNA gene sequencing microbiome assay. The genera that were significantly differential between positive and negative groups may serve as therapeutic targets for positive immune antibody-associated recurrent abortion.

## 1. Background

Spontaneous abortion is a pregnancy that spontaneously ends before the fetus can survive. The World Health Organization (WHO) defines this unsurvivable state as a fetus or embryo weighing 500 g or less, corresponding to a gestational age of 20–22 weeks or less [1]. Recurrent abortion is historically defined as three consecutive pregnancy losses in the first trimester (molar, ectopic, and biochemical pregnancies are not included) from the same biological father, which affects 5% of women of reproductive age [2].

Presently, there are a small number of accepted etiologies for recurrent abortions, such as genetic, anatomical, endocrine, immunological, and environmental factors [3]. Among these factors, immunological factors appear to be the most remarkable [4, 5]. During the last two decades, the relationships between autoantibodies and pregnancy loss have attracted extensive attention. The elevated plasma level of the antiphospholipid antibody has been mostly focused in recurrent abortion [6]. The antiphospholipid antibodies include different groups of autoantibodies that form against phospholipoproteins, including anticardiolipin (ACA), anti-beta2-glycoprotein 1 (GP), antiphosphatidylglycerol, lupus anticoagulant, antiphosphoserine, antiphosphatidylinositol, and antiphosphatidic acid [7–9]. In addition, antibodies to thyroid antigens, antinuclear antibodies (ANAs), antiprothrombin antibodies, and antilaminin have also been implicated in pregnancy complications [10–12].

Given that immunological aberrations may be the cause of recurrent abortion, several immunotherapies have been proposed to treat women with otherwise unexplained recurrent pregnancy loss [13]. Thereinto, the immunotherapy involving immunization with lymphocytes was considered and used previously, whereas there is still no consensus regarding the safety and efficacy of this therapy [2]. In China, this immunotherapy has been prohibited. Therefore, it is urgent to identify the underlying mechanisms of positive immune antibody-induced recurrent abortion so as to explore safe and effective therapies.

It has been reported that the commensal microbiota plays a central role in keeping immune homeostasis in health [14]. A recent study has demonstrated that gut microbiome changed obviously in some autoimmune disorders [15]. A recent study reported that vaginal microbiome was closely related with preterm birth [16]. We speculated that there may exist some relationships between the gut microbiome and positive immune antibody-associated recurrent abortion. Therefore, we assessed the gut microbiome composition in fecal samples from 20 recurrent abortion women with positive immune antibody and 20 with negative immune antibody using the 16s rRNA gene sequencing microbiome assay. Screening of pathogenic bacteria may advance the development of therapies that reverse the current trends in recurrent abortion.

## 2. Results

### 2.1. Data Processing

After sequencing, the raw reads for all samples ranged from 54,075 to 98,111. After quality control and removing the chimera sequences, the effective tags were obtained, and the effective rates ranged from 82.56% to 95.17%.

### 2.2. Operational Taxonomic Unit (OTU) Cluster and Annotation

**Figure 1:**
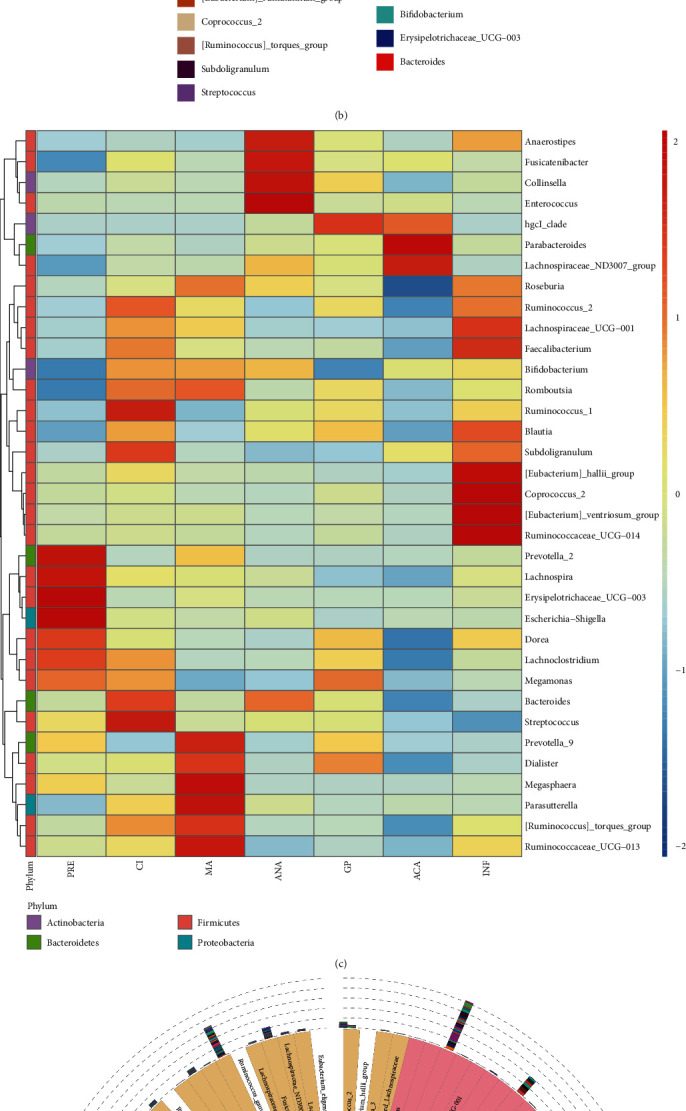
(a) The total tags, unique tags, taxon tags, unclassified tags, and operational taxonomic unit (OTU) number for each sample. (b) The relative abundance of species in the genus level (top 10). Others indicated the sum of relative abundance beyond the ten genera. (c) The heat map of species relative abundance. (d) The phylogenetic relationships of the representative sequences of top 100 genera.

The total tags, unique tags, taxon tags, unclassified tags, and OTU number for each sample are shown in Figure 1(a). The total numbers for the five indexes above were 2,610,543, 56,044, 2,554,499, 0, and 43,116, respectively. According to the results of OTU annotation, the top 10 maximum abundance of bacteria in each group in five taxonomic levels (phylum, class, order, family, and genus) were selected to generate the column accumulation graph of bacteria relative abundance. The relative abundance of bacteria in the genus level is shown in Figure 1(b). *Bacteroides* had the highest relative abundance in three subgroups of the positive group (11.25% for GP, 6.75% for ACA, and 14.96% for ANA). In the negative group, the genera with the highest relative abundance in cervical incompetence (CI), pregnancy at last (PRE), infertility (INF), and missed abortion (MA) were *Bacteroides* (16.12% for CI, 10.24% for PRE, 9.11% for INF, and 10.07% for MA), *Erysipelotrichaceae*_UCG-003 (0.74% for CI, 23.39% for PRE, 2.54% for INF, and 4.08% for MA), *Faecalibacterium* (10.92% for CI, 4.40% for PRE, 13.34% for INF, and 6.80% for MA), and *Prevotella*_9 (0.09% for CI, 7.55% for PRE, 0.92% for INF, and 15.21% for MA). Additionally, based on the annotation and abundance information of all subgroups at the genus level, the genera in the top 35 were selected for clustering analysis. The genera with top 3 higher abundance in PRE (*Erysipelotrichaceae*_UCG-003 (23.39%), *Bacteroides* (10.24%), and *Prevotella*_9 (7.55%)), CI (*Bacteroides* (16.12%), *Faecalibacterium* (10.92%), and *Blautia* (8.80%)), MA (*Prevotella*_9 (15.21%), *Bacteroides* (10.07%), and *Bifidobacterium* (8.26%)), INF (*Faecalibacterium* (13.34%), *Blautia* (9.90%), and *Bacteroides* (9.11%)), ANA (*Bacteroides* (14.96%), *Bifidobacterium* (7.89%), and *Blautia* (7.52%)), GP (*Bacteroides* (11.25%), *Blautia* (8.41%), and *Prevotella*_9 (7.73%)), and ACA (*Bacteroides* (6.75%), *Bifidobacterium* (6.25%), and *Blautia* (5.13%)) groups are shown in the clustering heat map (Figure 1(c)). The phylogenetic relationships of the representative sequences of top 100 genera were obtained through multiple sequence alignment, as shown in Figure 1(d).

### 2.3. Alpha Diversity Analysis

**Figure 2:**
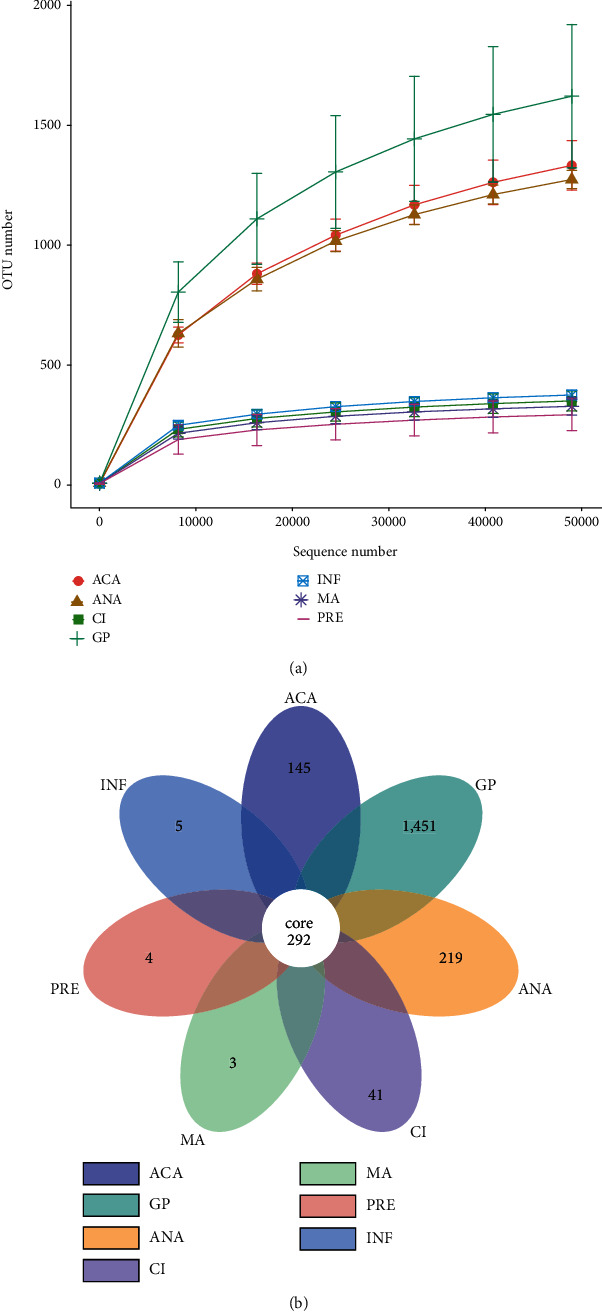
(a) Rarefaction curve for each subgroup. The *x*-axis is the number of sequencing bars randomly selected from a subgroup, and the *y*-axis is the number of operational taxonomic units (OTUs) constructed based on the number of sequencing bars. (b) The Venn diagram of OTUs in the seven groups. Different colors represent different subgroups.

The alpha diversity indexes for all groups are shown in Table 1. The results showed that the community richness, community diversity, and phylogenetic diversity in the positive group were higher than those in the negative group. Rarefaction curve is presented in Figure 2(a). The curves of each sample tended to flatten, suggesting that increasing sequencing depths did not help to discover new OTUs. The Venn diagram of OTUs in the seven groups is shown in Figure 2(b). There were 292 common OTUs in seven groups. The numbers of OTUs specific to ACA, GP, ANA, CI, MA, PRE, and INF were 145, 1,451, 219, 41, 3, 4, and 5, respectively.

### 2.4. Beta Diversity Analysis

**Figure 3:**
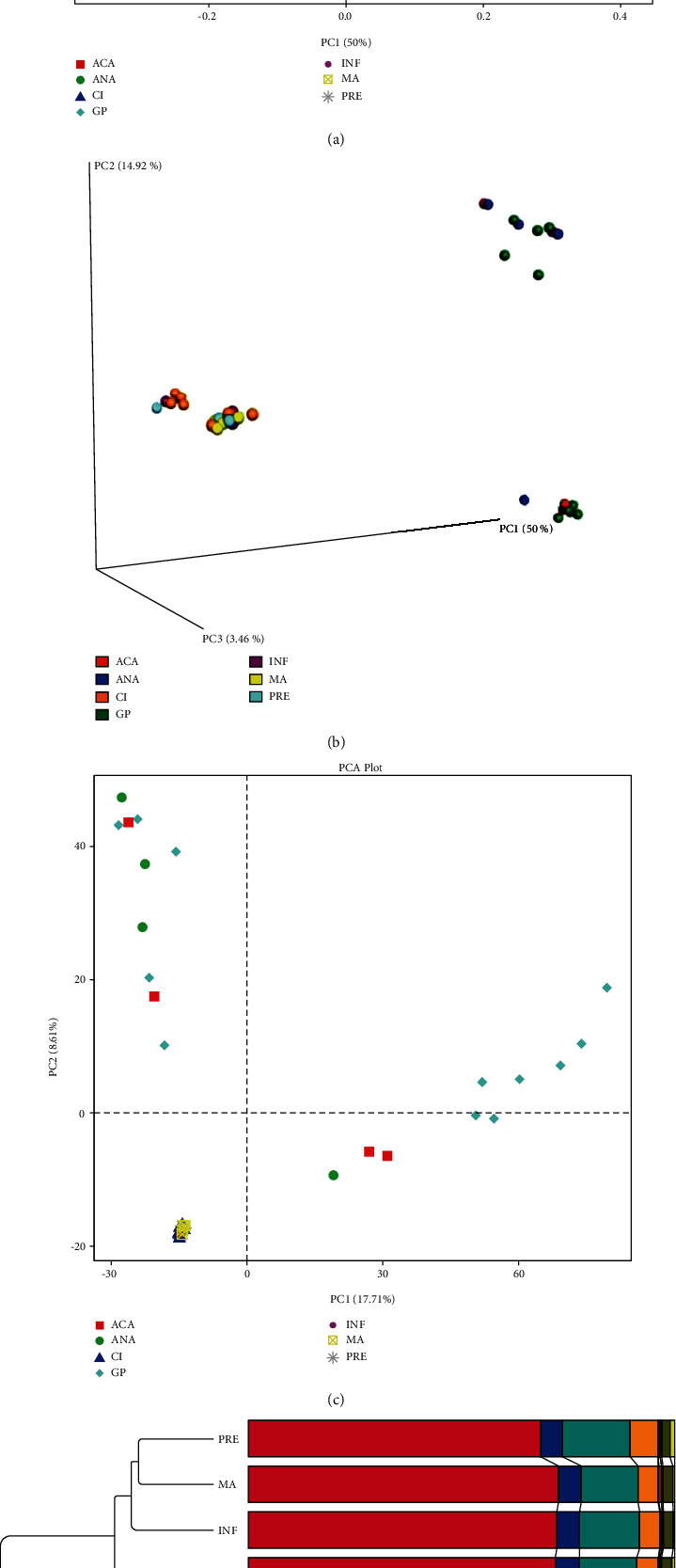
The results of PCoA (a, b) and PCA (c). The *x*-axis represents one principal component, the *y*-axis represents another principal component, and the percentage represents the contribution of the principal component to the sample difference. Each node represents a sample, and the samples from the same group are represented by the same color. (d) The UPGMA clustering tree in the phylum level.

Principal coordinate analysis (PCoA) was performed based on the unweighted UniFrac distance, as shown in Figures 3(a) and 3(b). The samples in the negative group tended to cluster together and were separated obviously from those in the positive group (PC1, which explained 50% of variation in the community). The principal component analysis (PCA) result showed that the community structure was distinct between positive and negative groups (PC1, which explained 17.73% of variation in the community) (Figure 3(c)). Additionally, the unweighted pair-group method with arithmetic means (UPGMA) clustering tree in the phylum level is shown in Figure 3(d). The three subgroups in the positive group were clustered together with Firmicutes having the highest relative abundance. Meanwhile, the four subgroups in the negative group were clustered together, and Proteobacteria had the highest relative abundance.

### 2.5. Analysis of Microbial Population Differences between Groups

**Figure 4:**
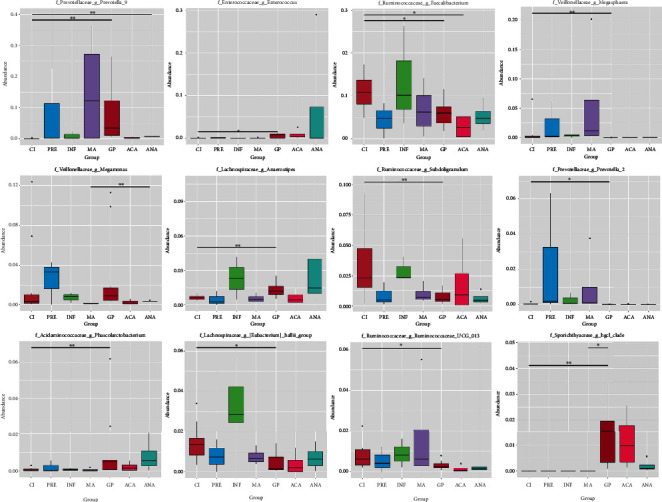
Statistical charts of significantly differential species between groups. The horizontal line represents the two groups with significant differences. ^∗^*q* value < 0.05, ^∗∗^*q* value < 0.01.

The Metastat method was used to identify the microbial population with significant differences among seven subgroups. The microbial populations with significant differences were screened according to the *q* value, and the abundance distribution boxes of these bacteria (top 12) in the genus level are shown in Figure 4, such as *Prevotella*_9 (CI vs. GP; CI vs. ANA), *Enterococcus* (CI vs. GP), *Faecalibacterium* (CI vs. GP; CI vs. ACA), *Megasphaera* (CI vs. GP), and *Megamonas* (MA vs. ANA).

**Figure 5:**
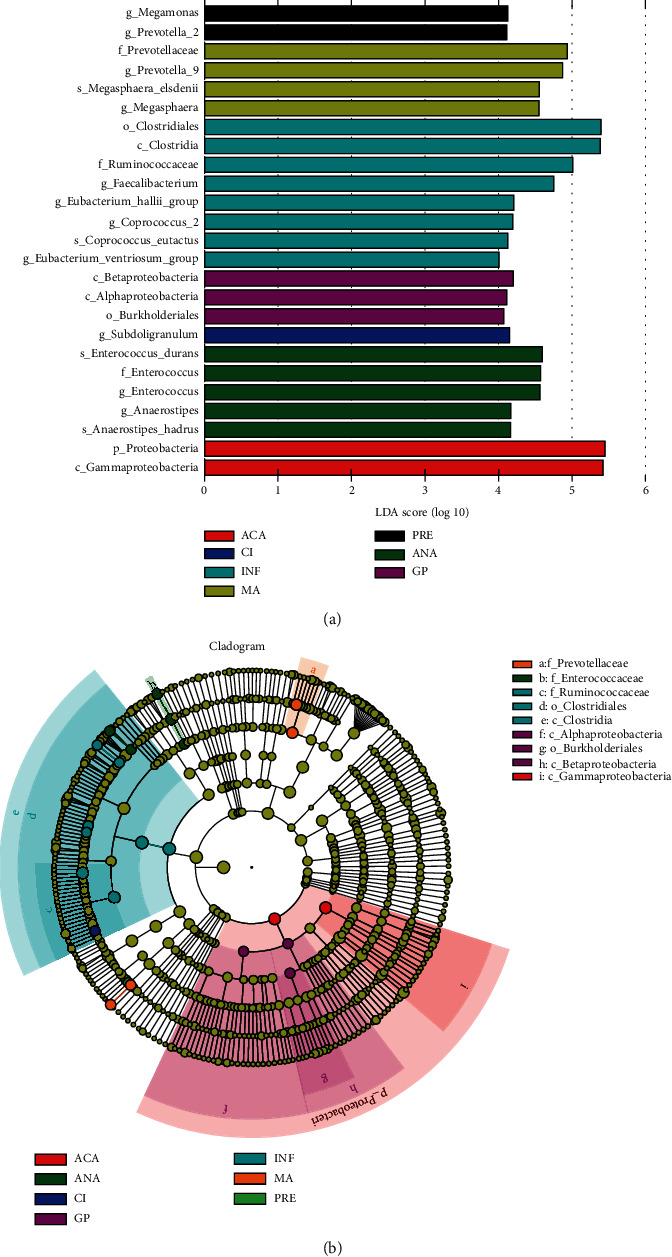
LDA value distribution histogram (a) and evolutionary branching diagram (b). LDA value distribution histogram shows the species whose LDA score is greater than the set value (the default setting is 4). In an evolutionary bifurcation diagram, a circle radiating from inside to outside represents the taxonomy level from phylum to the genus (or species). Each small circle at different taxonomy levels represents a taxonomy under that level, and the size of the small circle is proportional to the relative abundance. Coloring principle: yellow represents no significant difference. The significantly differential species is colored according to the group.

LEfSe analysis is used to search biomarkers with statistical differences between groups [17]. As shown in Figure 5, *Megamonas* and *Prevotella*_2 were significantly differential genera in the PRE subgroup; *Prevotella*_9 and *Megasphaera* were significantly differential genera in the MA subgroup; *Megasphaera*, *Faecalibacterium*, *Eubacterium*_*hallii*_group, *Coprococcus*_2, and *Eubacterium*_*ventriosum*_group were significantly differential species in INF; *Subdoligranulum* was significantly differential genera in CI. In the positive group, *Enterococcus* and *Anaerostipes* were significantly differential genera in the ANA subgroup; Burkholderiales was a significantly differential order in GP; and Gammaproteobacteria was a significantly differential class in ACA.

## 3. Discussion

With the development of the next-generation sequencing technology, the gut microbiota has been suggested to be associated with the promotion of health as well as the initiation or maintenance of gastrointestinal and nongastrointestinal diseases [18]. However, the role of gut microbiota in positive immune antibody-associated recurrent abortion has not been investigated to our best knowledge.

In the present study, a total of 43,116 OTUs were obtained in all 40 samples. OTU annotation revealed that *Bacteroides* had the highest relative abundance in all three subgroups of the positive group. In the negative group, the genera with the highest relative abundance in CI, PRE, INF, and MA were *Bacteroides*, *Erysipelotrichaceae*_UCG-003, *Faecalibacterium*, and *Prevotella*_9, respectively. It is noteworthy that *Bacteroides* had a high relative abundance in both positive and negative groups. *Bacteroides* is a genus of Gram-negative, obligate anaerobic bacteria [19]. *Bacteroides* constitutes enterotype 1 as well as the most common dominance inside gut microbiota in the healthy person [20]. A recent study has reported that *Bacteroides* spp. are part of normal placental microbiome [21]. However, the existence of a normal placental microbiome has not been confirmed by all authors. For instance, Leiby et al. [22] investigated possible placenta colonization associated with spontaneous preterm birth and found no consistent microbial signature unique to placenta in term or preterm births. A recent study reported that *Bacteroides fragilis* was involved in gynecological infections [23], which was implicated with spontaneous midgestation abortion and premature rupture of membrane [24, 25].

In addition to *Bacteroides* and *Blautia* also had a higher relative abundance in the positive group and two subgroups of the negative groups. *Blautia* is a genus of anaerobic, Gram-positive bacteria found in the gut. A study has reported that the overweight pregnant women with greater serum zonulin have higher abundance of *Bacteroides* and *Blautia* [26]. Previous studies have reported that obesity may increase the risk of sporadic abortion and recurrent abortion in pregnancy conceived spontaneously [27, 28]. Nevertheless, *Blautia* has been reported to have a controversial role in the human gut. Some studies reported an association between *Blautia* and hyperglycemia [29, 30], while other studies revealed that the abundance of *Blautia* indicated a healthy gut and reduced risk for type 1 diabetes and obesity [31, 32]. Given the high abundance of *Blautia* in feces of recurrent abortion women, we speculated that this taxon may be implicated in recurrent abortion.

Alpha diversity analysis revealed that the community richness, community diversity, and phylogenetic diversity in the positive group were higher than those in the negative group, suggesting that there may exist some correlation between immune antibodies (ACA, ANA, and GP) of recurrent abortion women and community diversity of gut microbiota, whereas, in contrast with our study, Wei et al. [33] recently reported lower rarefaction curves in the case of autoimmune hepatitis. Thus, further study needs to be done to explore the potential mechanisms.

To further analyze the gut microbiota involved in positive immune antibody-induced recurrent abortion, significantly differential genera between positive and negative groups were identified through Metastat and LEfSe analyses. *Prevotella*_9, *Enterococcus*, and *Megasphaera* presented significant differences between CI (negative) and GP (positive) subgroups. *Prevotella*_9 was also a significantly different genus between CI and ANA. Moreover, *Enterococcus* and *Anaerostipes* were significant biomarkers in the ANA subgroup. A species of *Prevotella*, *Prevotella bivia*, is reported to be a frequent bacterial species in bacterial vaginosis, which is associated with intra-amniotic infections and an increased risk of preterm birth [34]. Importantly, Graham et al. has suggested a relationship between the abortion and the bacterial vaginosis [35]. Among *Enterococcus* sp., *Enterococcus faecalis* is a ubiquitous Gram-positive bacterium that occurs widely in the vagina and alimentary tract [36]. Increasing evidence suggests that *Enterococcus faecalis* could pass through placental barriers and cause adverse outcomes during pregnancy [37]. Additionally, a study has reported that *Megasphaera* sp. is significantly associated with abortion. Women with *Megasphaera* sp. infection had a higher risk of getting multiple abortions than those without such infection [38]. *Anaerostipes* represents over 2% of the total colonic microbiota in the healthy colon [39]. Furthermore, they are suggested to play an important role in the gut ecosystem given their ability to produce butyrate from lactate [40, 41], whereas their roles in recurrent abortion have not been reported. Taken together, we speculated that *Prevotella*_9, *Enterococcus*, *Megasphaera*, and *Anaerostipes* may serve as therapeutic targets for positive immune antibody-associated recurrent abortion.

Nevertheless, in this study, the sequences with ≥97% similarity were assigned to the same OTUs, which had some limits and may constitute a possible bias. Additionally, the results in this study were not validated. Thus, further validation experiments are needed to confirm our findings.

## 4. Conclusions

In conclusion, the present study for the first time investigated the gut microbiome involved in positive immune antibody-associated recurrent abortion via the 16s rRNA gene sequencing microbiome assay. Some genera with high abundance, such as *Bacteroides* and *Blautia*, may be implicated in recurrent abortion. Additionally, the genera that were significantly differential between positive and negative groups may serve as therapeutic targets for positive immune antibody-associated recurrent abortion.

## 5. Methods

### 5.1. Subjects and Fecal Sample Collection

A total of 40 women with recurrent abortions, including 20 cases who were positive for immune antibody (ACA, ANA, and GP; positive group) and 20 cases negative for the antibody (negative group), were included in this study. In the negative group, there were four types of cases: PRE, CI, MA, and INF. Specifically, INF referred to the woman who was negative for the antibodies, had a history of multiple miscarriages, had been diagnosed with recurrent abortion, and was not pregnant at the time of fecal sample collection. Those who meet any of the following conditions were excluded: (1) with a history of gastric and intestinal resection, vomiting, constipation, or other digestive diseases; (2) with a history of malignancy and underlying medical conditions, including diabetes, liver disease, or cardiopulmonary disease; (3) with a history of mental illness, mobility difficulties, communication barriers, etc.; (4) with a history of long-term use of specific drugs, drug abuse, smoking, or alcoholism; (5) there is a history of use of antibiotics or microecological preparations four weeks before feces collection; (6) minorities with special dietary habits; and (7) with infectious diseases, such as hepatitis B, hepatitis C, syphilis, and AIDS. The statistical comparison of baseline information (age, weight, height, systolic/diastolic blood pressure, body mass index (BMI), and abortion history) in two groups is shown in Table 2. Fecal samples of 40 subjects were collected and stored at -80°C within 2 h.

This study was approved by the medical ethics committee of Qilu Hospital, Shandong University. All of the participants had given the informed written consent prior to their participation.

### 5.2. Genome DNA Extraction and Amplicon Generation

Total genome DNA was extracted from all samples using the cetyltrimethylammonium bromide and sodium dodecyl sulfate method. After detection of the concentration and purity of the DNA on 1% agarose gels, DNA was diluted with sterile water to 1 ng/*μ*L. The 16s rRNA amplicons covering variable region V4 were amplified using a specific primer (16S V4: 515F-806R) with the barcode. PCR reaction was conducted with a Phusion® High-Fidelity PCR Master Mix (New England Biolabs, Beverly, MA, USA). The PCR products were quantified with electrophoresis on 2% agarose gel and then mixed in equidensity ratios and purified with Qiagen Gel Extraction Kit (Qiagen, Hilden, Germany).

### 5.3. Library Preparation and Sequencing

Sequencing libraries were constructed using a TruSeq® DNA PCR-Free Sample Preparation Kit (Illumina, San Diego, CA, USA), and the quality of libraries was evaluated on the Qubit® 2.0 Fluorometer (Thermo Scientific, MA, USA) and Agilent Bioanalyzer 2100 system. Following that, the libraries were sequenced on the Illumina HiSeq 2500 platform, and 250 bp paired-end reads were produced.

The raw data have been deposited in NCBI-SRA database (https://www.ncbi.nlm.nih.gov/sra/PRJNA505198).

### 5.4. Assembly and Quality Control of the Paired-End Reads

Paired-end reads were assigned to the samples according to their unique barcode and were truncated by cutting off the barcode and primer sequence. These paired-end reads were merged into raw tags using FLASH (V1.2.7) [42]. The raw tags underwent quality filtering to select the high-quality clean tags [43] using QIIME (V1.7.0) [44]. These clean tags were then compared with the reference database (Gold database, http://drive5.com/uchime/uchime_download.html) using the UCHIME algorithm [36], followed by removal of the chimera sequences [45] to obtain the effective tags.

### 5.5. OTU Cluster and Annotation

All effective tags were clustered by the UPARSE software (V7.0.1001) [46]. The sequences with ≥97% similarity were assigned to the same OTUs. The representative sequences for OTUs were selected for annotation of taxonomic information using the Mothur method and the SSU rRNA database [47] in SILVA [48].

### 5.6. Phylogenetic Relationship Construction

A multiple sequence alignment was conducted to study the phylogenetic relationship of different OTUs and the differences of the dominant species in different groups using the MUSCLE software (V3.8.31) [49].

### 5.7. Data Normalization

The abundance information of OTUs was normalized by using a standard of sequence number (cutoff = 34778) corresponding to the sample with the least sequences. The alpha diversity and beta diversity analyses were performed based on these normalized data.

### 5.8. Alpha Diversity Analysis

Alpha diversity is used to analyze the complexity of species diversity for a sample via seven indices, including Chao1, ACE, Shannon, Simpson, Good_coverage, Observed_species, and PD_whole_tree. These indices were calculated using QIIME (V1.7.0) and displayed with R software (V2.15.3). Two indices, Chao (the Chao1 estimator) and ACE (the ACE estimator), were used to identify the community richness. Shannon (the Shannon) and Simpson (the Simpson indices) were used to identify the community diversity. Coverage (the Good_coverage) was used to characterize the sequencing depth. PD_whole_tree (PD_whole_tree index) was used to identify phylogenetic diversity.

### 5.9. Beta Diversity Analysis

Beta diversity analysis was used to evaluate the differences of different samples in species complexity. Beta diversity on unweighted UniFrac was calculated by the QIIME software to construct a UPGMA sample clustering tree. Cluster analysis was conducted by PCA using the ade4 package and ggplot2 package in R software (V2.15.3). PCoA was conducted to get principal coordinates and visualized from complex multidimensional data, which was displayed by stat, WGCNA, and ggplot2 packages in R software (V2.15.3).

### 5.10. Analysis of Species Differences between Groups

LDA effect size (LEfSe) was conducted using LEfSe software with LDA score of 4. Metastat analysis was performed using R software at all taxonomy levels. The *p* value was obtained through a permutation test, which was adjusted using the Benjamin and Hochberg false discovery rate to obtain the *q* value [50].

## Figures and Tables

**Table 1:** The alpha diversity indexes for all groups.

Sample name	Observed_species	Shannon	Simpson	Chao1	ACE	Good_coverage	PD_whole_tree
CI.1	261	4.856	0.938	290.4	300.761	0.999	24.464
CI.2	350	5.086	0.932	388.077	390.784	0.999	28.009
CI.3	366	5.933	0.97	413.275	419.862	0.999	28.174
CI.4	411	5.574	0.959	453.6	465.297	0.999	31.004
CI.5	381	5.949	0.969	410.018	425.252	0.999	30.173
CI.6	349	4.144	0.799	393.279	419.659	0.998	29.19
CI.7	372	5.842	0.961	392.788	401.884	0.999	31.751
CI.8	294	3.355	0.717	315.522	326.958	0.999	24.271
CI.9	376	6.06	0.972	401.021	409.373	0.999	29.959
CI.10	349	5.743	0.957	379.447	394.602	0.999	28.167
PRE.1	200	1.693	0.484	225.738	237.462	0.999	20.744
PRE.2	343	5.475	0.945	395.286	394.285	0.999	27.875
PRE.3	337	5.675	0.96	375.897	370.499	0.999	27.248
INF.1	349	5.468	0.952	388.396	398.279	0.999	27.324
INF.2	396	6.007	0.965	431.019	440.331	0.999	30.886
INF.3	382	6.089	0.969	420.038	441.474	0.999	29.723
MA.1	381	5.342	0.939	418.344	439.417	0.999	28.602
MA.2	341	5.851	0.964	356	367.947	0.999	27.052
MA.3	311	4.164	0.832	349.278	351.205	0.999	25.795
MA.4	281	4.344	0.898	302	309.143	0.999	24.459
GP.1	1945	7.394	0.975	2224.504	2278.574	0.991	140.89
GP.2	2045	7.099	0.967	2420.34	2471.05	0.989	148.199
GP.3	1780	6.819	0.958	2024.215	2144.297	0.991	128.873
GP.4	1749	6.606	0.933	2018.777	2097.36	0.991	130.84
GP.5	1926	7.062	0.968	2188.081	2332.944	0.99	138.375
GP.6	1678	6.837	0.96	1934.121	1979.725	0.992	124.303
GP.7	1852	7.174	0.97	2094.881	2207.793	0.991	135.121
GP.8	1255	6.341	0.948	1453.527	1562.637	0.993	108.539
GP.9	1323	6.824	0.966	1382.922	1439.513	0.996	107.276
GP.10	1370	6.101	0.923	1696.166	1731.909	0.992	113.333
GP.11	1435	7.286	0.981	1651.679	1668.09	0.993	115.359
GP.12	1096	6.807	0.977	1243.027	1315.791	0.995	94.035
ACA.1	1366	2.7	0.396	1619.696	1687.878	0.992	108.788
ACA.2	1487	2.72	0.392	1739.207	1883.191	0.991	117.454
ACA.3	1249	5.667	0.926	1627.76	1683.128	0.991	110.188
ACA.4	1229	6.914	0.973	1410.166	1444.161	0.994	102.885
ANA.1	1208	5.207	0.889	1431.372	1476.76	0.993	95.491
ANA.2	1291	5.871	0.943	1520.728	1635.309	0.992	112.395
ANA.3	1294	6.77	0.952	1472.909	1518.464	0.994	105.233
ANA.4	1303	6.589	0.969	1525.107	1584.558	0.993	115.884

**Table 2:** Comparison of baseline information between the two groups.

Characteristic	Positive group	Negative group	*t*/*Z*	*p* value
Age	32.35 ± 4.21	30.90 ± 4.20	1.090	0.283
Height	161.20 ± 4.51	161.15 ± 5.37	0.032	0.975
Systolic blood pressure	114.50 ± 10.68	121.65 ± 14.17	-1.802	0.079
Diastolic blood pressure	70.55 ± 7.39	70.25 ± 7.92	0.124	0.902
BMI	24.12 ± 4.10	28.71 ± 7.01	-2.525	0.017
Weight	60.00 (55.50, 65.00)^∗^	73.75 (56.25, 88.00)^∗^	-1.883	0.060
Abortion history	2 (2, 2)^∗^	2 (2, 3)^∗^	-1.023	0.306

## Data Availability

The data used to support the findings of this study are available from the corresponding author upon request.
